# Strand-specific miR-28-3p and miR-28-5p have differential effects on nasopharyngeal cancer cells proliferation, apoptosis, migration and invasion

**DOI:** 10.1186/s12935-019-0915-x

**Published:** 2019-07-19

**Authors:** Yan Lv, Huijun Yang, Xingkai Ma, Geping Wu

**Affiliations:** 1 Center of Translational Medicine, The Affiliated Zhangjiagang Hospital of Soochow University, No. 68, Jiyang West Road, Suzhou, China; 2 Department of Otolaryngology, The Affiliated Zhangjiagang Hospital of Soochow University, No. 68, Jiyang West Road, Suzhou, China

**Keywords:** miR-28-3p, miR-28-5p, Nasopharyngeal cancer, Proliferation, Invasion

## Abstract

**Background:**

MicroRNAs (miRNAs) play crucial roles in varieties of cancers, particularly in tumorigenesis, progression, and migration. Dysregulation of miR-28 was reported to occur in various types of human malignancies. In humans, two different mature miRNA sequences are excised from opposite arms of the stem-loop pre-miR-28, hsa-miR-28-3p and hsamiR-28-5p. However, the expression and distinct role of miR-28-3p and miR-28-5p in nasopharyngeal carcinoma (NPC) remain undetermined.

**Methods:**

The expressions of miR-28-3p/-5p in human NPC tissues were tested by quantitative real-time PCR. miR-28-3p/-5p were overexpressed by mimics and silenced by inhibitors. The roles of miR-28-3p/-5p in NPC development were studied using cultured HONE-1 cells.

**Results:**

The mRNA expression levels of miR-28-3p and -5p were significantly decreased in NPC tissues in comparison with adjacent normal tissues. Overexpression of miR-28-5p suppressed NPC cell proliferation and induced cell cycle arrest and apoptosis, while miR-28-3p promoted NPC cell migration and invasion. The miRNAs effected on different signal pathways: miR-28-5p altered expression of cyclin D1 and influenced the PI3K/AKT signaling pathway. In contrast, miR-28-3p downregulated Nm23-H1 and accelerated the process of EMT.

**Conclusion:**

miR-28-3p and -5p were both downregulated in NPC tissues but had distinct biological effects in NPC cells. They may serve as potential prognostic markers and therapeutic targets for NPC.

## Background

Nasopharyngeal carcinoma (NPC) is a highly malignant disease that originates in the nasopharynx epithelium. It has a particularly high incidence in Southern China, Southeast Asia and North Africa [1]. Many studies have shown that NPC is a complex disease that can be attributed to the interactions between Epstein–Barr virus (EBV) infection, environmental factors, and genetic susceptibility [2]. Although NPC is radiation sensitive, the results of patients with advanced stages of disease are unsatisfactory due to chemoresistance, relapse, and distant metastasis [3–5]. As a result, the 5-year survival rate of NPC is less than 60% [6]. However, little is known about the exact genetic changes in the pathogenesis of NPC [7]. Therefore, it is necessary to study the molecular mechanisms underlying the progression of NPC to improve the prognosis.

MicroRNAs (miRNAs) are highly conserved, endogenous small noncoding RNAs with ~ 20 nucleotides, that disturb gene expression by binding to the 3′ untranslated region (3′UTR) of their target mRNAs [8]. It can regulate gene expression at the translational or post-transcriptional level. It does not have an open reading frame (ORF) and does not encode proteins. Accumulating evidence suggests that miRNAs play important roles in various types of cancers and are involved in tumor occurrence and development [9]. miRNAs can function as either oncogenes or tumor suppressor genes depending on the type of tumor or the cellular context [10]. miRNAs play crucial roles in NPC tumorigenesis and may serve as potential biomarkers and therapeutic targets [11–13]. However, the function of miRNAs in NPC cells is still largely unexplored.

miRNAs are transcribed from a gene locus in the genome to generate pri-miRNAs with 5′ cap and 3′ polyA tail [14]. Then processed into pre-miRNA by Drosha/DGCR8, and transported to the cytoplasm via Exportin-5 [15]. Pre-miRNA undergoes further cleavage by Dicer to form mature miRNA [16]. In some cases, two mature miRNAs can be excised from the same stem-loop pre-miRNA [17]. These "5p" and "3p" miRNAs are biologically different in terms of stability and functionality. In humans, two mature miRNAs, miR-28-3p and miR-28-5p, are derived from 3′ and 5′ ends of pre-miR-28, respectively. miR-28-3p and miR-28-5p targets several cancer-related genes and is hence involved in cell proliferation, migration, invasion and epithelial–mesenchymal transition (EMT) [18–22]. However, the expression and biological function of these miRNAs in NPC remain largely undefined.

In this study, we discovered that miR-28-3p and miR-28-5p were both downregulated in NPC tissues but had distinct biological effects in NPC cells. miR-28-5p inhibited NPC cell proliferation, and induced apoptosis and cell cycle arrest but had no effect on cell migration and invasion in vitro. In contrast, miR-28-3p accelerated the migration and invasion abilities of NPC cell but had no significant influence on cell proliferation and apoptosis.

## Materials and methods

### NPC tissue specimens

Total human NPC samples were obtained from 30 patients at the Affiliated Zhangjiagang Hospital of Soochow University (Suzhou, China). This study was approved by the Human Research Ethics Committee of the Soochow University, and informed consent was obtained from each patient.

### Cell line

Human NPC cell line, HONE-1, were cultured with RPMI 1640 medium RPMI-1640 medium (GIBCO, Grand Island, USA) supplemented with 100 U/ml penicillin–streptomycin solution and 10% fetal bovine serum (FBS, Gibco, NY, USA), in a 37 °C incubator containing 5% CO_2_. The detailed protocol was described in detail in our previous studies [23]. HONE-1 was acquired from the Institute of Cell Biology at the Chinese Academy of Sciences (Shanghai, China).

### Cell transfection

miR-28-3p/-5p mimic, inhibitor and the negative control were designed by RiboBio (Guangzhou, China). miRNA transfection was performed using riboFECT™ CP Reagent (RiboBio, Guangzhou, China) according to the manufacturer’s instructions. After 24, 48, 72, 96 h of transfection, the transfected cells were harvested for miRNAs or total RNA isolation, and protein extraction.

### Real-time quantitative polymerase chain reaction (qRT-PCR)

The detailed protocol was described in our previous studies [24–26]. The qRT-PCR reactions were performed on an ABI 7500 system (Applied Biosystems, USA) with SYBR Green PCR Master Mixes (Thermo Fisher). A human U6 small nuclear RNA was used for normalization. Primer sequences are as follows:

miR-28-3p-Forward: CGCGCACTAGATTGTGAGCT;

miR-28-3p-Reverse: AGTGCAGGGTCCGAGGTATT;

miR-28-3p-RT: GTCGTATCCAGTGCAGGGTCCGAGGTATTCGCACTGGATACGACTCCAGG; miR-28-5p-Forward: GCGCATTGCACTTGTCTCG;

miR-28-5p-Reverse: AGTGCAGGGTCCGAGGTATT;

miR-28-5p-RT: GTCGTATCCAGTGCAGGGTCCGAGGTATTCGCACTGGATACGACTCAGAC; U6-Forward: CTCGCTTCGGCAGCACA;

U6-Reverse: AACGCTTCACGAATTTGCGT.

### Western blotting assay

Western blot analysis was carried out as previously described [24–26]. Following antibodies were utilized: p-Akt (9271, Cell Signaling Technology, 1:1000 dilution), Akt (9272, Cell Signaling Technology, 1:1000 dilution), Cyclin D1 (60186-1-Ig, Proteintech, 1:2000 dilution), Nm23-H1 (sc-514515, Santa Cruz, 1:500 dilution), Ecadherin (20874-1-AP, Proteintech, 1:2000 dilution) and GAPDH (60004-1-Ig, Proteintech, 1:10,000 dilution). Secondary antibodies (A0208, HRP-labeled Goat Anti-Rabbit IgG, Beyotime, 1:1000 dilution; A0216, HRP-labeled Goat Anti-Mouse IgG, Beyotime, 1:1000 dilution) were used.

### Cell viability assay

HONE-1 cells were seeded at a density of 3000 cells per well in 96-well plates and transfected with miR-28-3p/-5p mimic, inhibitor and the negative control. After incubation for 0, 24, 48 and 72 h, 10 μl cell counting assay kit-8 solution (Dojindo, Japan) was added to all wells and incubated at 37 °C for another 2 h. Optical absorbance of each well at 450 nm was measured with a microplate reader (Bio-Rad Laboratories, USA).

### Colony formation assay

Cells were plated in 6-well plates at low density (3 × 10^3^ cells/well) and cultured for 8 days. Cells were then washed with PBS and stained with crystal violet. The number of clones re-generated (colonies > 50 cells each) was scored under a microscope.

### EdU assay

EdU Apollo®488 In Vitro Imaging Kit (Ribo Bio, China) was used to evaluate the proliferation of HONE-1 cells. In according to the manufacturer’s advice, 5-ethynyl-20-deoxyuridine (EdU) with a final concentration of 50 mM was added and the cells were incubated at 37 °C for 2 h. Cell nucleus were stained with Hoechst for 15–30 min and visualized by a fluorescent microscope (Leica, DM 4000, Germany).

### Cell cycle distribution analysis

Cells were harvested at 24 h and fixed by ice-cold ethanol at 4 °C overnight, and then stained with PI/RNase staining buffer (BD Biosciences, China) for 30 min at room temperature. DNA content was measured by using a Navios Flow Cytometer (Beckman Coulter, Brea, CA, USA).

### Cell apoptosis analysis

Cells were collected 48 h after transfection. For apoptosis analysis, cells were double stained using FITC Annexin V Apoptosis Detection Kit I (BD Pharmingen, 556547). After that, cells were detected by a Navios Flow Cytometer (Beckman Coulter, Brea, CA, USA) in 1 h.

### Trypan blue staining

Trypan blue staining cell survival assay kit (Beyotime, Nantong, China) was used to test cell death rate: the collected cells are resuspended with appropriate cell resuspension solution according to the number of cells. Add 0.1 ml of cell suspension to 0.1 ml Trypan blue solution (2x), mix gently and stain for 3 min, then count with a hemocytometer.

### Wound healing assay

Cells were grown in a 6-well plate. After infection, cell layers were scratched with a plastic tip to form a straight line and washed twice with PBS solution. Under a light microscope, cells were imaged at 0 h and 24 h time points to observe the wound healing process. 1.0 μg/ml mitomycin (Sigma) was always added to exclude the influence of cell proliferation.

### In vitro cell migration and invasion assays

Cell migration capabilities were detected by using Transwell chambers (Corning, New York, NY) as previously described [25]. Transwell chambers were pre-coated with 1 mg/ml Matrigel (BD Biosciences, Shanghai, China) and then used to test cell invasion abilities. 1.0 μg/ml mitomycin (Sigma) was always added to exclude the influence of cell proliferation.

### Statistical analysis

All data were analyzed by the SPSS 20.0 analysis. The data shown in this study were obtained at least three independent experiments and all results represent the means ± S.D. The comparison between two groups was performed by the Student's t-test. Comparison of more than two groups was performed by one-way ANOVA. Statistical significance was defined as *P < 0.05, **P < 0.01, ***P < 0.001.

## Result

### miR-28-3p and miR-28-5p are downregulated in human NPC tissues

**Fig. 1:**
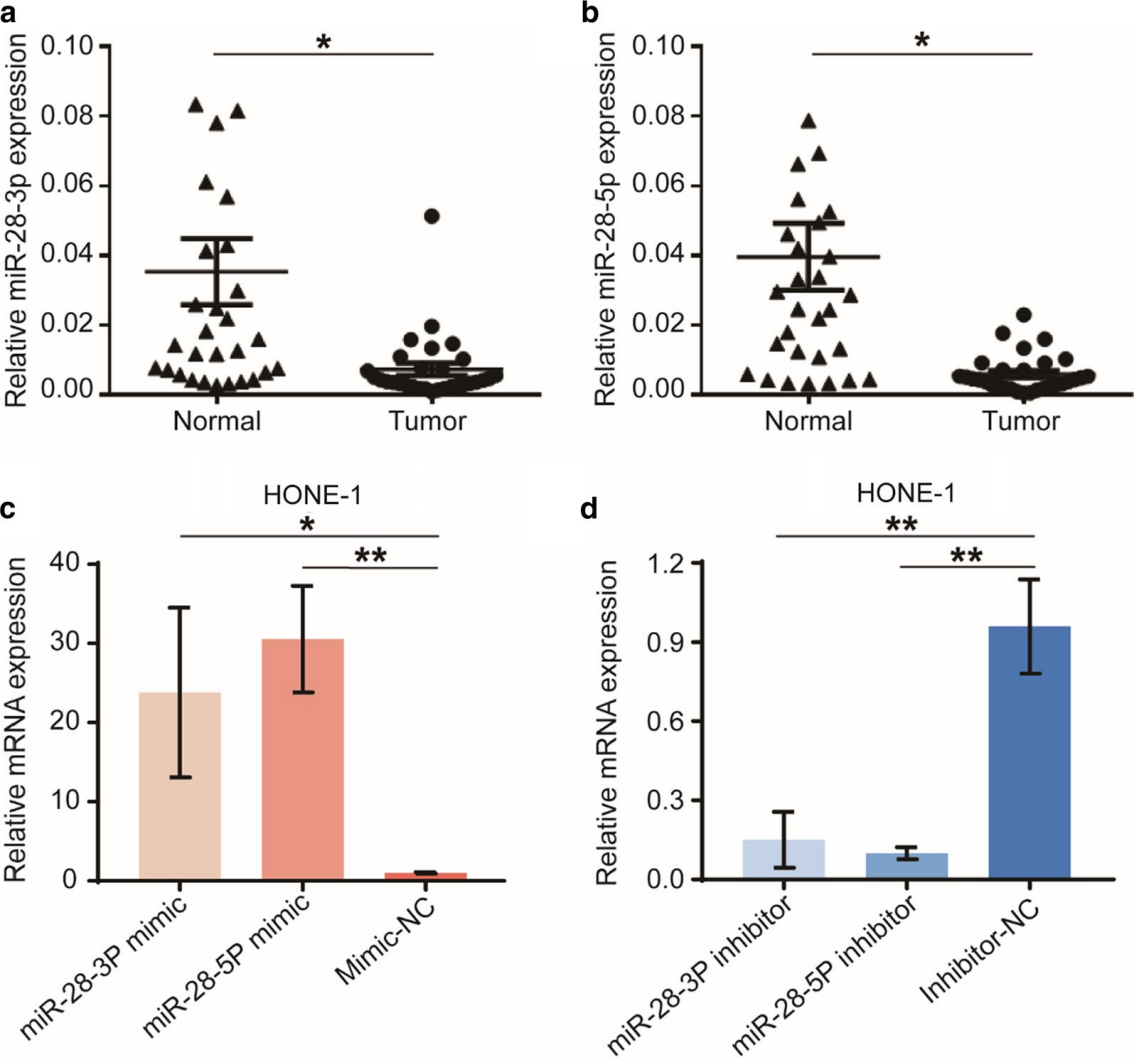
Expression of miR-28-3p and miR-28-5p in human NPC tissues. The mRNA expression of miR-28-3p (**a**) and miR-28-5p (**b**) in NPC (“Tumor”) and paired surrounding normal tissues (“Normal”, n = 30) were examined by qRT-PCR. Short double-stranded RNAs (miRNA mimics) and their OMe-modified antisense oligonucleotides (miRNA inhibitors) were used to overexpress (**c**) and knockdown (**d**) miR-28-3p and miR-28-5p in HONE-1 cells. *P < 0.05, **P < 0.01, vs “Normal” samples or negative control

To evaluate the role of miR-28-3p/-5p in the development of NPC, the expression levels of them were measured in 30 pairs of NPC tumor tissues and paired adjacent normal tissues. The results in Fig. 1a, b showed that the NPC samples had a significantly lower level of miR-28-3p/-5p expression compared with adjacent normal tissue (P < 0.05). These data suggest that miR-28-3p and miR-28-5p are both downregulated in NPC.

### miR-28-5p suppresses NPC cell proliferation and induces cell cycle arrest in vitro

To investigate the potential functions of miRNAs in NPC cell behaviors, we performed overexpression and knockdown experiments by direct transfection of short double-stranded RNAs (miRNA mimics) and the OMe-modified antisense oligonucleotides (miRNA inhibitors). Transfection efficiency was detected using real-time quantitative PCR. Relative to mimic-negative control-transfected cells, transfection of the miR-28-3p-mimic and miR-28-5p-mimic in HONE-1 cells significantly increased mRNA expression levels of miR-28-3p and miR-28-5p (Fig. 1c). Besides, transfection of the miR-28-3p-inhibitor and miR-28-5p-inhibitor significantly decreased miR-28-3p and miR-28-5p levels compared with inhibitor-negative control (Fig. 1d).

**Fig. 2:**
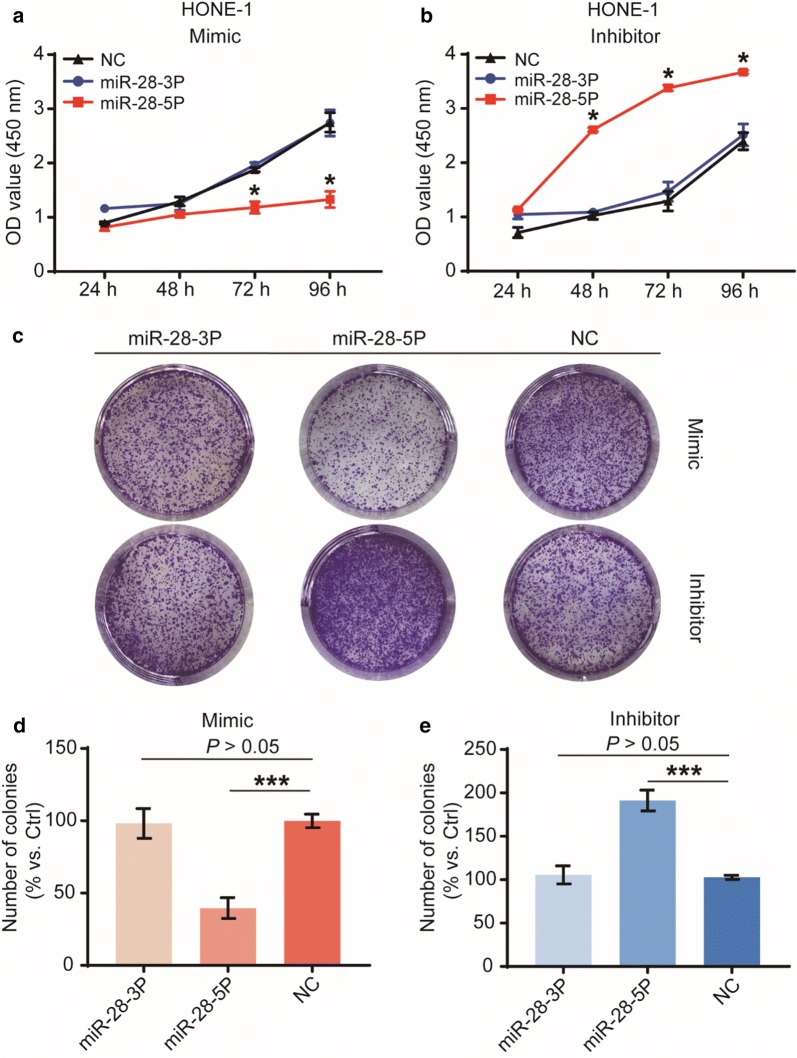
Biological functions of miR-28-3p and miR-28-5p in cell viability and cell growth in vitro. Cell viability was determined by CCK-8 assay (**a**, **b**). The effect of miR-28-3p/-5p on tumorigenic capability of the NPC cells was measured by the colony-forming assay (**c**), Number of colonies were counted at the 7th day after transfection with mimics (**d**) and inhibitors (**e**) of miR-28-3p/-5p. The data are representative of three repeats. *P < 0.05, ***P < 0.001, vs negative control

**Fig. 3:**
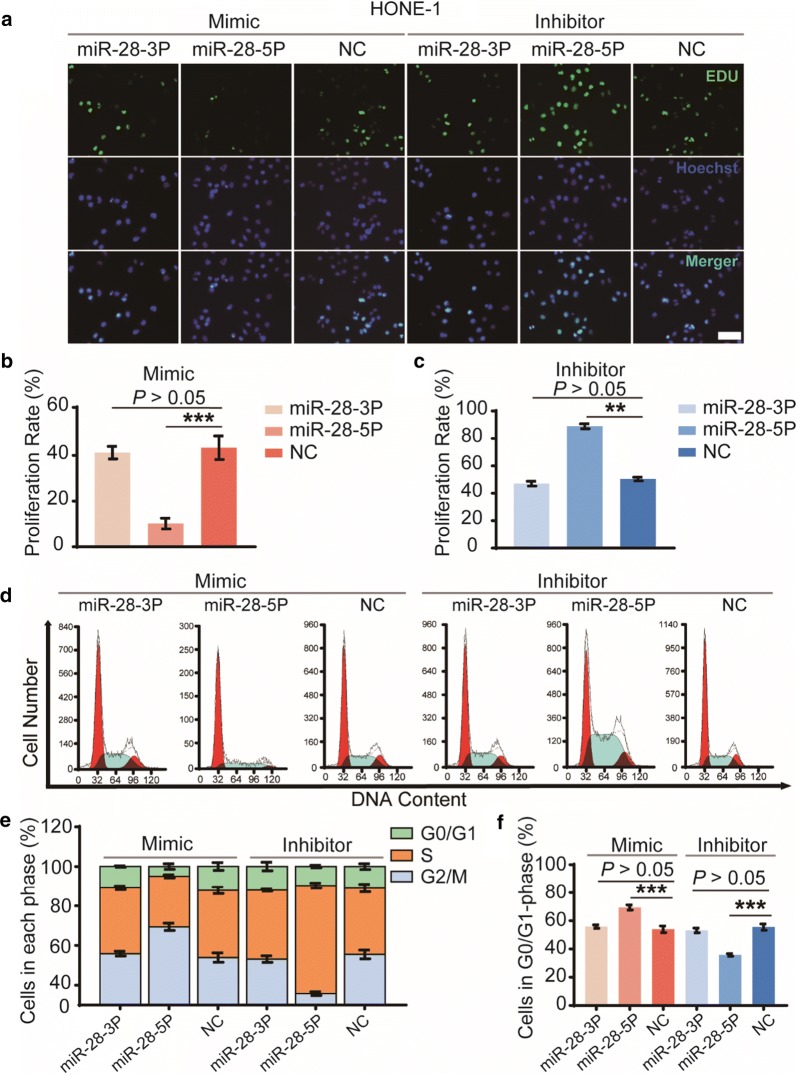
Roles of miR-28-3p and miR-28-5p in cell proliferation and cell-cycle progression in vitro. Cell proliferation was determined using EdU assay at 48 h after transfection (**a**); Number of EdU positive staining cells were counted, and cell proliferation rate was calculated after transfection with mimics (**b**) and inhibitors (**c**) of miR-28-5p/-3p and negative control. The cell-cycle progression was analyzed by PI-FACS assay staining at 48 h (**d**). Cell cycle was divided into G0/G1, S and G2/M, and cell cycle distribution was counted respectively (**e**). Cells in G0/G1-phase were detected (**f**). The data represent three independent experimental repeats. **P < 0.01, ***P < 0.001, vs negative control. Scale bar = 100 μm

To evaluate the effects of miR-28-3p/-5p on the NPC cell viability and cell proliferation, we performed the Cell Counting Kit-8 (CCK-8) viability assays, colony formation assays, EDU assays and cell cycle distribution analysis. Results suggested that overexpression of miR-28-5p significantly inhibited HONE-1 cell viability (Fig. 2a, b), reduced colony number (Fig. 2c–e) and EDU staining (Fig. 3a–c), moreover, induced G1-phase arrest (Fig. 3d, e). In contrast, knockdown of miR-28-5p has the opposite effect. In HONE-1 cells overexpressing or knockdown miR-28-3p has no statistically significant differences at any time compared with cells transfected with negative controls. These results indicated that miR-28-5p, but not miR-28-3p, suppresses NPC cell proliferation and induces cell cycle arrest in vitro.

### miR-28-5p induces NPC cell apoptosis in vitro

**Fig. 4:**
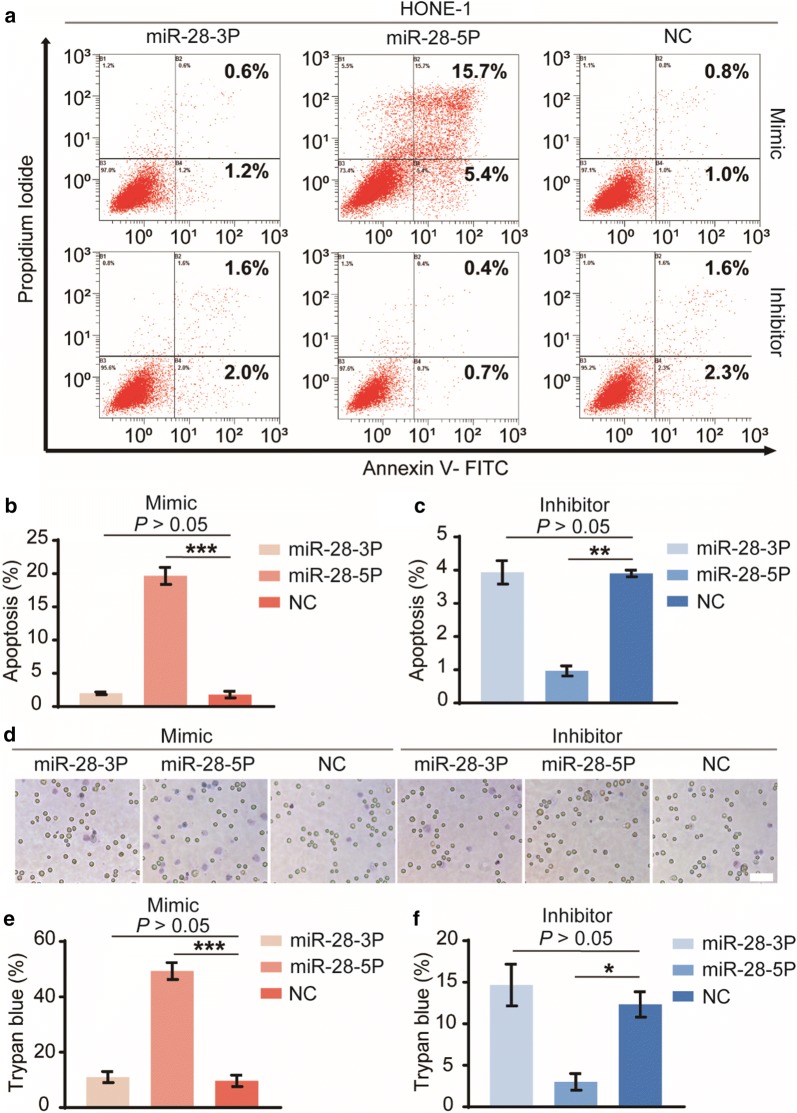
Effects of miR-28-3p and miR-28-5p in cell apoptosis and death. The apoptosis of HONE-1 cells was examined by Annexin V-FITC/PI staining and analyzed by flow cytometry after transfection with mimics and inhibitors of miR-28-5p/-3p and negative control for 96 h (**a**). The early and late stages of apoptotic cells were all counted for cell apoptosis (**b**, **c**). Additionally, cell death was examined by trypan blue staining (**d**). The number of dying cells (trypan blue positive cells) were detected (**e**, **f**). The data are representative of three repeats. *P < 0.05, **P < 0.01, ***P < 0.001, vs negative control. Scale bar = 100 μm

The cell apoptosis was examined by Annexin V-FITC/PI staining and analyzed by flow cytometry. Results demonstrated that overexpression of miR-28-5p significantly increased Annexin V staining, a marker for early stages of apoptosis (Fig. 4a, b). Knockdown of miR-28-5p reduced Annexin V staining observably (Fig. 4a, c). Likewise, trypan blue staining revealed that, compared with the negative control groups, the number of dying cells (trypan blue positive cells) increased in the miR-28-5p mimic group (Fig. 4d, e), but decreased in the miR-28-5p inhibitor group (Fig. 4d, f). miR-28-3p had no effect in the above experiments. These data together suggest that miR-28-5p, but not miR-28-3p, lead to HONE-1 cell apoptosis and death in vitro.

### miR-28-3p promotes NPC cell migration and invasion in vitro

**Fig. 5:**
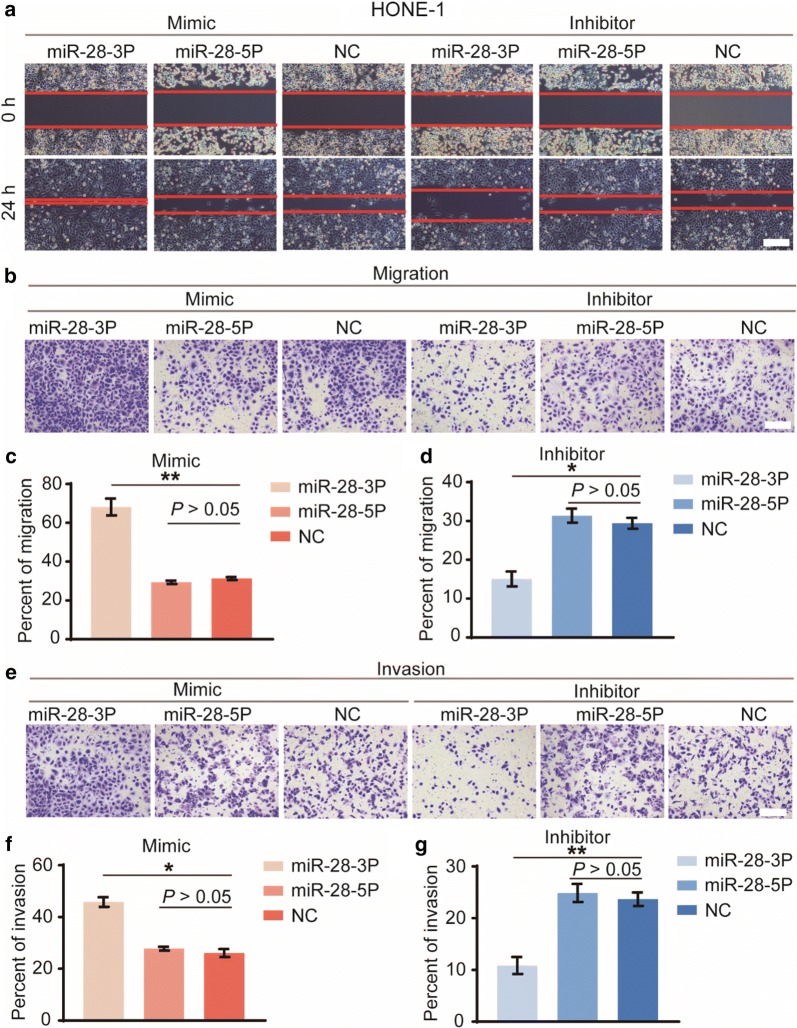
Biological effects of miR-28-3p and miR-28-5p in cell migration and invasion. In vitro cell migration was tested by the wound healing assay (**a**). We also used the transwell cell migration assays to explore NPC cell migratory capacity (**b**). Cells that migrated into the lower well were stained, photographed and counted (**c**, **d**). Invasion assay was carried out in transwell chambers with matrigel-coated (**e**). Cells that invaded into the lower well were counted (**f**, **g**). All assays were performed in triplicate. *P < 0.05, **P < 0.01, vs negative control. Scale bar = 200 μm

To further understand the functions of miR-28-3p/-5p in NPC metastasis, we performed the wound healing assay and transwell cell migration assays. Results of the wound healing assay showed that HONE-1 cells transfected with miR-28-3p mimic displayed increased migration ability as compared with the mimic negative control group (Fig. 5a). In contrast, cells transfected with miR-28-3p inhibitor displayed decreased migration ability compared with the inhibitor negative control group. However, there were no statistically significant differences obtained for miR-28-5p mimic or inhibitor groups compared with the negative controls. Further, the same result was obtained when using the transwell cell migration assays to explore migratory capacity (Fig. 5b–d).

To evaluate the effects of miR-28-3p/-5p on invasive ability of tumor cells, we performed the invasion assay using the Matrigel Invasion Chamber. The same trend was observed that forced miR-28-3p expression significantly increased the invasive ability of cells (Fig. 5e, f) whereas knockdown of miR-28-3p led to a significant reduction in cell invasion compared with the negative controls (Fig. 5e, g). There were no significant differences obtained for miR-28-5p mimic or inhibitor groups compared with the negative controls. Taken together, these data demonstrated that miR-28-3p, but not miR-28-5p, promotes NPC cell migration and invasion in vitro.

### miR-28-3p and miR-28-5p regulate different signaling pathways

The above results suggested that miR-28-5p inhibits tumorigenesis by restraining cell proliferation and inducing cell cycle arrest and cell apoptosis in vitro, but it has no effect on cell migration and invasion. In contrast, miR-28-3p promotes NPC cell migration and invasion, but has no effect on cell proliferation and apoptosis. To identify signaling pathways that could be involved in the different biological effects caused by these miRNAs, we used western blot to detect changes at the protein level for several tumor proliferation, apoptosis and migration-related proteins in cells transfected with miR-28-3p/-5p mimics, inhibitors and negative controls.

**Fig. 6:**
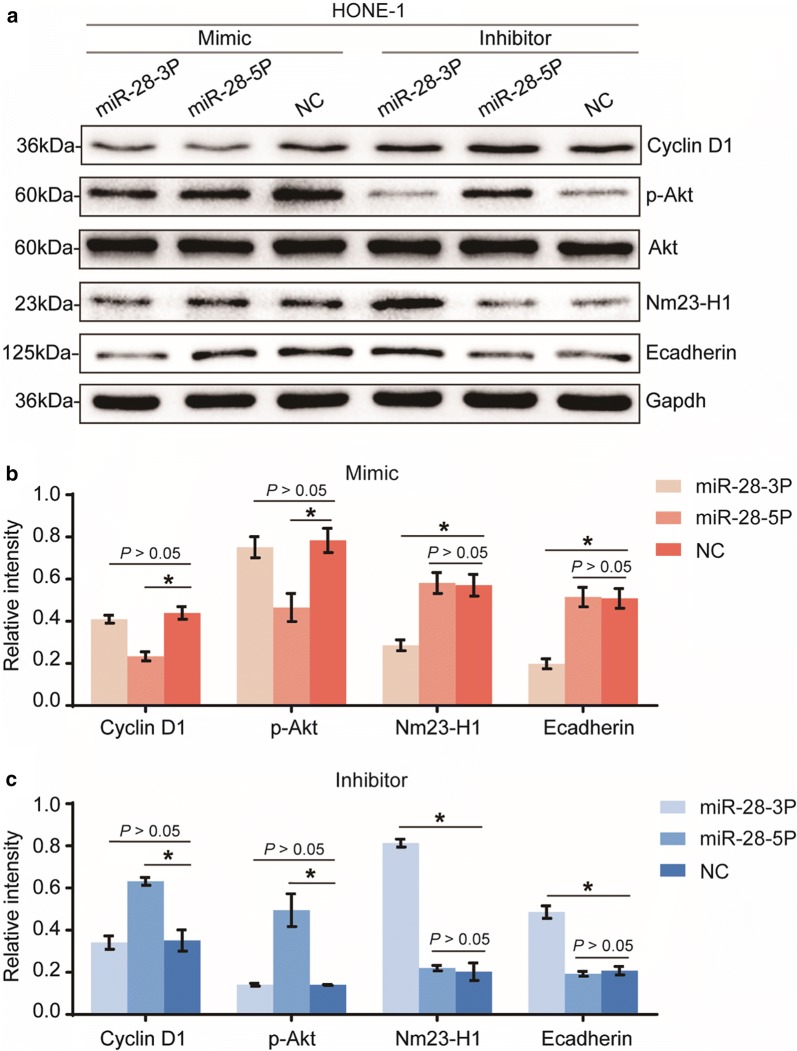
miR-28-3p and miR-28-5p mediate the malignant biological behavior of NPC cells via different signaling pathways. Western blot analysis of p-Akt, Cyclin D1, Nm23-H1 and Ecadherin levels in cells transfected with mimics and inhibitors of miR-28-3p/-5p and negative control (**a**). Qualification of the data shown in **a** (**b**, **c**). All assays were performed in triplicate. *P < 0.05, vs negative control

Previous studies have suggested that miR-28-5p altered expression of Cyclin D1 whereas miR-28-3p bound Nm23-H1 [18], we then tested the effect of miR-28-5p/-3p overexpression and knockdown on the expression of the above proteins. Besides, we also tested some key factors of different signaling pathways involved in tumor proliferation, apoptosis and migration, such as, PI3K/AKT pathway and EMT. Our results confirmed that overexpression of miR-28-5p downregulated Cyclin D1, p-Akt protein expression (Fig. 6a, b). In contrast, suppression of miR-28-5p upregulated Cyclin D1, p-Akt protein expression (Fig. 6a, c). However, miR-28-5p has no effect on the regulation of Nm23-H1 and Ecadherin protein expression (Fig. 6a–c). As expected, overexpression of miR-28-3p downregulated Nm23-H1 and Ecadherin protein expression (Fig. 6a, b), whereas suppression of miR-28-3p upregulated Nm23-H1, Ecadherin protein expression (Fig. 6a, c). However, miR-28-3p has no effect on the regulation of Cyclin D1 and p-Akt protein expression.

Taken together, these data consistently suggest that miR-28-5p suppresses NPC cell proliferation, induces cell cycle arrest and apoptosis by downregulating the expression of the oncogenes, such as Cyclin D1 and p-Akt. However, miR-28-3p promotes NPC cell migration and invasion by regulating the expression of genes such as Nm23-H1 and Ecadherin. This may explain, at least in part, the observed different biological effects of miR-28-3p and miR-28-5p.

## Discussion

Identifying specific miRNAs in tumors and their targets is critical for understanding their role in tumorigenesis and progression and may help to explore new therapeutic targets [12]. miRNAs may function either as oncogenes or tumor suppressors and are heavily involved in the progression and metastasis of solid tumors [27]. The significance of change in the expression of miRNAs that could serve as diagnostic and therapeutic biomarkers in NPC has been emphasized [11, 28, 29].

Studies have reported miR-28 to be frequently dysregulated in several types of cancers such as colorectal cancers [18, 22], renal cell carcinoma [19], Lymphoma [20] and ovarian cancers [21]. However, a limited number of studies have examined the functions and mechanisms of miR-28 involved in progression and metastasis. Only 1 study has addressed the distinct functions of the miR-28 hairpin RNA products, miR-28-3p and miR-28-5p, Maria et al. demonstrated that overexpression of miR-28-5p reduced CRC cell proliferation, migration and invasion in vitro, whereas miR-28-3p increased CRC cell migration and invasion [18].

In this study, we report that expression of miR-28-3p and miR-28-5p are downregulated in NPC tissues. Additionally, we observed the distinct roles of miR-28-3p and miR-28-5p in malignant biological behavior of NPC cells, such as cell growth, proliferation, apoptosis resistance, migration and invasion. We found that miR-28-5p, but not miR-28-3p, suppresses NPC cell proliferation and induces cell cycle arrest and apoptosis in vitro. In contrast, miR-28-3p, but not miR-28-5p, promotes NPC cell migration and invasion. Most of our results are consistent with previous studies, but we have found that miR-28-5p has no effect on NPC cell migration and invasion in vitro, when mitomycin is used to exclude the influence of cell proliferation.

miRNAs may act as tumor suppressors or oncogenes depending on whether their specific targets act as oncogenes or tumor suppressors [27]. Previous studies have shown that miR-28-3p and miR-28-5p regulate different signaling pathways during tumorigenesis and development. miR-28-5p altered expression of *CCND1* in CRC [18] and may suppress PI3K/AKT by inhibiting IGF1 expression in HCC [30]. Cyclin D1, encoded by the *CCND1* gene, is a well-known oncogene that is overexpressed in several types of tumors [31, 32], including NPC [33]. This protein is a key player in cell-cycle regulation [34]. PI3K/AKT pathway is involved in the regulation of cell cycle progression and cellular growth [35]. In line with these findings, our present study showed that overexpression of miR-28-5p, but not miR-28-3p, decreased cyclin D1 and p-Akt protein level in HONE-1 cells. The results indicate that miR-28-5p acts as a tumor suppressor in NPC.

miR-28-3p has the capacity of regulating *NM23-H1* [18], the first metastasis-suppressor gene identified [36]. Our present study showed that overexpression of miR-28-3p, but not miR-28-5p, decreased Nm23-H1 protein level in HONE-1 cells. These findings are consistent with previous studies. Interestingly, we found that miR-28-3p can regulate the expression of E cadherin, which is the most important marker in the process of epithelial–mesenchymal transition (EMT) [37]. The adhesion between epithelial cells mediated by E cadherin is an effective way to inhibit tumor cell invasion and metastasis [38]. In summary, these findings manifest that miR-28-3p promotes NPC cell migration and invasion in vitro, at least in part by inhibiting Nm23-H1 and E cadherin expression.

## Conclusion

Taken together, the results demonstrated that the miR-28 hairpin RNA products, miR-28-3p and miR-28-5p, are significantly downregulated in NPC cells, but they have distinct biological effects. miR-28-5p inhibits the tumorigenesis of NPC by downregulating Cyclin D1 and influencing the activation of the PI3K/AKT signaling pathway. In contrast, miR-28-3p promotes the invasion and metastasis of NPC cell by downregulating Nm23-H1 and accelerate the process of EMT. Such information has a crucial application for the design of microRNA gene therapy trials.

## Data Availability

All data generated or analyzed during this study are included in this published article.

## Abbreviations

NPC: nasopharyngeal carcinoma
EMT: epithelial–mesenchymal transition
PI3K: phosphoinositide 3-kinase
AKT: also known as Protein Kinase B

## References

[1] Chua MLK, Wee JTS, Hui EP, Chan ATC (2016). Nasopharyngeal carcinoma. Lancet.

[2] Huang SCM, Tsao SW, Tsang CM (2018). Interplay of viral infection, host cell factors and tumor microenvironment in the pathogenesis of nasopharyngeal carcinoma. Cancers (Basel).

[3] Lee AW, Ma BB, Ng WT, Chan AT (2015). Management of nasopharyngeal carcinoma: current practice and future perspective. J Clin Oncol.

[4] Genova P, Brunetti F, Bequignon E, Landi F, Lizzi V, Esposito F, Charpy C, Calderaro J, Azoulay D (2016). de'Angelis N: Solitary splenic metastasis from nasopharyngeal carcinoma: a case report and systematic review of the literature. World J Surg Oncol.

[5] Prawira A, Oosting SF, Chen TW, Delos Santos KA, Saluja R, Wang L, Siu LL, Chan KKW, Hansen AR (2017). Systemic therapies for recurrent or metastatic nasopharyngeal carcinoma: a systematic review. Br J Cancer.

[6] Lan M, Chen C, Huang Y, Tian L, Duan Z, Han F, Liao J, Deng M, Sio TT, Prayongrat A (2017). Neoadjuvant chemotherapy followed by concurrent chemoradiotherapy versus concurrent chemoradiotherapy alone in nasopharyngeal carcinoma patients with cervical nodal necrosis. Sci Rep.

[7] Bruce JP, Yip K, Bratman SV, Ito E, Liu FF (2015). Nasopharyngeal cancer: molecular landscape. J Clin Oncol.

[8] Calin GA, Croce CM (2006). MicroRNA signatures in human cancers. Nat Rev Cancer.

[9] To KK (2013). MicroRNA: a prognostic biomarker and a possible druggable target for circumventing multidrug resistance in cancer chemotherapy. J Biomed Sci.

[10] Croce CM (2009). Causes and consequences of microRNA dysregulation in cancer. Nat Rev Genet.

[11] Lee KT, Tan JK, Lam AK, Gan SY (2016). MicroRNAs serving as potential biomarkers and therapeutic targets in nasopharyngeal carcinoma: a critical review. Crit Rev Oncol Hematol.

[12] Lin CH, Chiang MC, Chen YJ (2018). MicroRNA-328 inhibits migration and epithelial–mesenchymal transition by targeting CD44 in nasopharyngeal carcinoma cells. Onco Targets Ther.

[13] Wang LX, Kang ZP, Yang ZC, Ma RX, Tan Y, Peng XB, Dai RZ, Li J, Yu Y, Xu M (2018). MicroRNA-135a inhibits nasopharyngeal carcinoma cell proliferation through targeting interleukin-17. Cell Physiol Biochem.

[14] Lee Y, Jeon K, Lee JT, Kim S, Kim VN (2002). MicroRNA maturation: stepwise processing and subcellular localization. EMBO J.

[15] Lee Y, Ahn C, Han J, Choi H, Kim J, Yim J, Lee J, Provost P, Radmark O, Kim S (2003). The nuclear RNase III Drosha initiates microRNA processing. Nature.

[16] Kim VN (2005). MicroRNA biogenesis: coordinated cropping and dicing. Nat Rev Mol Cell Biol.

[17] Griffiths-Jones S, Grocock RJ, van Dongen S, Bateman A, Enright AJ (2006). miRBase: microRNA sequences, targets and gene nomenclature. Nucleic Acids Res.

[18] Almeida MI, Nicoloso MS, Zeng L, Ivan C, Spizzo R, Gafa R, Xiao L, Zhang X, Vannini I, Fanini F (2012). Strand-specific miR-28-5p and miR-28-3p have distinct effects in colorectal cancer cells. Gastroenterology.

[19] Wang C, Wu C, Yang Q, Ding M, Zhong J, Zhang CY, Ge J, Wang J, Zhang C (2016). miR-28-5p acts as a tumor suppressor in renal cell carcinoma for multiple antitumor effects by targeting RAP1B. Oncotarget.

[20] Bartolome-Izquierdo N, de Yebenes VG, Alvarez-Prado AF, Mur SM, Lopez Del Olmo JA, Roa S, Vazquez J, Ramiro AR (2017). miR-28 regulates the germinal center reaction and blocks tumor growth in preclinical models of non-Hodgkin lymphoma. Blood.

[21] Xu J, Jiang N, Shi H, Zhao S, Yao S, Shen H (2017). [Corrigendum] miR-28-5p promotes the development and progression of ovarian cancer through inhibition of N4BP1. Int J Oncol.

[22] Tsiakanikas P, Kontos CK, Kerimis D, Papadopoulos IN, Scorilas A (2018). High microRNA-28-5p expression in colorectal adenocarcinoma predicts short-term relapse of node-negative patients and poor overall survival of patients with non-metastatic disease. Clin Chem Lab Med.

[23] Wang SS, Lv Y, Xu XC, Zuo Y, Song Y, Wu GP, Lu PH, Zhang ZQ, Chen MB (2019). Triptonide inhibits human nasopharyngeal carcinoma cell growth via disrupting Lnc-RNA THOR-IGF2BP1 signaling. Cancer Lett.

[24] Lv Y, Si M, Chen N, Li Y, Ma X, Yang H, Zhang L, Zhu H, Xu GY, Wu GP (2017). TBX2 over-expression promotes nasopharyngeal cancer cell proliferation and invasion. Oncotarget.

[25] Lv Y, Song Y, Ni C, Wang S, Chen Z, Shi X, Jiang Q, Cao C, Zuo Y (2018). Overexpression of lymphocyte antigen 6 complex, locus e in gastric cancer promotes cancer cell growth and metastasis. Cell Physiol Biochem.

[26] Zuo Y, Lv Y, Qian X, Wang S, Chen Z, Jiang Q, Cao C, Song Y (2018). Inhibition of HHIP promoter methylation suppresses human gastric cancer cell proliferation and migration. Cell Physiol Biochem.

[27] Zhang B, Pan X, Cobb GP, Anderson TA (2007). microRNAs as oncogenes and tumor suppressors. Dev Biol.

[28] Chen HC, Chen GH, Chen YH, Liao WL, Liu CY, Chang KP, Chang YS, Chen SJ (2009). MicroRNA deregulation and pathway alterations in nasopharyngeal carcinoma. Br J Cancer.

[29] Wang LJ, Chou YF, Chen PR, Su B, Hsu YC, Chang CH, Lee JW (2014). Differential miRNA expression in repeated recurrence of nasopharyngeal carcinoma. Cancer Lett.

[30] Shi X, Teng F (2015). Down-regulated miR-28-5p in human hepatocellular carcinoma correlated with tumor proliferation and migration by targeting insulin-like growth factor-1 (IGF-1). Mol Cell Biochem.

[31] Musgrove EA, Caldon CE, Barraclough J, Stone A, Sutherland RL (2011). Cyclin D as a therapeutic target in cancer. Nat Rev Cancer.

[32] Jirawatnotai S, Hu Y, Livingston DM, Sicinski P (2012). Proteomic identification of a direct role for cyclin d1 in DNA damage repair. Cancer Res.

[33] Tsang CM, Yip YL, Lo KW, Deng W, To KF, Hau PM, Lau VM, Takada K, Lui VW, Lung ML (2012). Cyclin D1 overexpression supports stable EBV infection in nasopharyngeal epithelial cells. Proc Natl Acad Sci USA.

[34] Nishi K, Inoue H, Schnier JB, Rice RH (2009). Cyclin D1 downregulation is important for permanent cell cycle exit and initiation of differentiation induced by anchorage-deprivation in human keratinocytes. J Cell Biochem.

[35] Fresno Vara JA, Casado E, de Castro J, Cejas P, Belda-Iniesta C, Gonzalez-Baron M (2004). PI3K/Akt signalling pathway and cancer. Cancer Treat Rev.

[36] Steeg PS, Bevilacqua G, Pozzatti R, Liotta LA, Sobel ME (1988). Altered expression of NM23, a gene associated with low tumor metastatic potential, during adenovirus 2 Ela inhibition of experimental metastasis. Cancer Res.

[37] Kalluri R, Weinberg RA (2009). The basics of epithelial–mesenchymal transition. J Clin Invest.

[38] Canel M, Serrels A, Frame MC, Brunton VG (2013). E-cadherin-integrin crosstalk in cancer invasion and metastasis. J Cell Sci.

